# Microstructural characterization of near-surface microstructures on rail wheels in service – an insight into “stratified surface layers”

**DOI:** 10.12688/openreseurope.15881.2

**Published:** 2023-07-03

**Authors:** Matthias Freisinger, Andreas Trausmuth

**Affiliations:** 1 AC2T research GmbH, Wiener Neustadt, Austria

**Keywords:** Wheel-rail contact, White Etching Layer, Brown Etching Layer, Stratified Surface Layer

## Abstract

**Background:** To decrease maintenance costs and improve safety in rail transportation, the understanding of rail and wheel defects is vital. Studies on “white etching layers” (WEL) on rails and wheels, prone to fatigue crack initiation, have been extensively studied. Recently, a relative named “brown etching layer” (BEL) and its combination, the so-called “stratified surface layer” (SSL), are observed in the field. This study presents an investigation on a rail wheel affected by mechanical and thermal loadings from service with focus on the different evolved layers in the near-surface region.

**Methods: **Optical microscopy is performed on etched cross-sectional cuts to identify different evolved microstructures (WEL, BEL, SSL), further, specific regions are investigated in detail by scanning electron microscopy to evaluate the microstructural characteristics.  To analyze the change in mechanical properties, low-load Vickers hardness investigations are executed in distinctive zones.

**Results: **This study highlights the broad variety of evolved microstructures, however, a rough classification of WEL (fine mesh-like microstructure, 900 – 1200 HV0.01) and BEL (globular cementite particles, 400 – 600 HV0.01) is given. Further, results indicate that the BEL is commonly accompanied by a WEL, representing an SSL.

**Conclusions: **The complex loading situation in a wheel-rail contact can lead to the formation of WEL, BEL and SSL. The observation of numerous initiated fatigue cracks within these regions demonstrates the relevance of in-depth studies on evolved microstructures in wheel-rail contacts.

## Introduction

Material defects on rails and wheels are the main cause of maintenance costs in rail traffic ^1^. With increasing train frequency in recent years, increased wear and degradation of rails and wheels are expected, which emphasizes the importance of understanding rail and wheel defects to ensure safety and reliability of rail transportation. The terminology of rail and wheel defects is diverse and has been the subject of numerous studies in recent decades ^1– 4^. However, it is evident that high mechanical and thermal loads affect the near-surface microstructure in a significant way, leading to a microstructure evolution over time in service, depending on the loading history experienced ^5– 7^. A well-known microstructure found on rail and wheel surfaces is the so-called “white etching layer” (WEL), named after its white appearance under optical microscopy when etching with an ethanol nitric acid. Its formation is described either mechanically by severe plastic deformation and/or thermally by increased temperature and rapid cooling ^8– 11^. WELs are related to the stud defects on rails and presumed to affect the initiation of rolling contact fatigue clusters in wheels ^2, 12– 16^. In rolling-sliding contacts, WELs are commonly described as martensitic microstructure with thicknesses from several micrometers up to several hundreds of micrometers, high hardness values (700 – 1000 HV) and low fracture toughness ^9, 17– 21^. In recent studies a related near-surface microstructure evolved in wheel-rail contact is observed, the so-called “brown etching layer” (BEL) ^22– 25^, coming up with a brownish appearance under an optical microscope. The concurrent observation of WEL and BEL pictures a stratification of the near-surface microstructure, hence, the name “stratified surface layer” (SSL) is introduced ^25^. However, not many studies are done on BELs, especially on wheel samples. Due to the gradual wear of rails and wheels and the unknown local loading history of rail and wheel samples from the field, as well as the further influence of the degree of etching on the staining of the microstructure, a clear definition of WEL or BEL is rarely possible.

Within this work, we detected variations of WELs, BELs, and SSLs on an ER7 wheel from service with a milage of ~200,000 km. The evolved near-surface microstructures are characterized to improve the understanding of specific evolved near-surface microstructures on rail wheels, with focus on the less studied BEL. The aim of this work is to point out the variety of mechanically and thermally affected microstructures on rail wheels to improve the knowledge which can contribute to the reduction of maintenance costs in rail transportation.

## Methods

**Figure 1.**
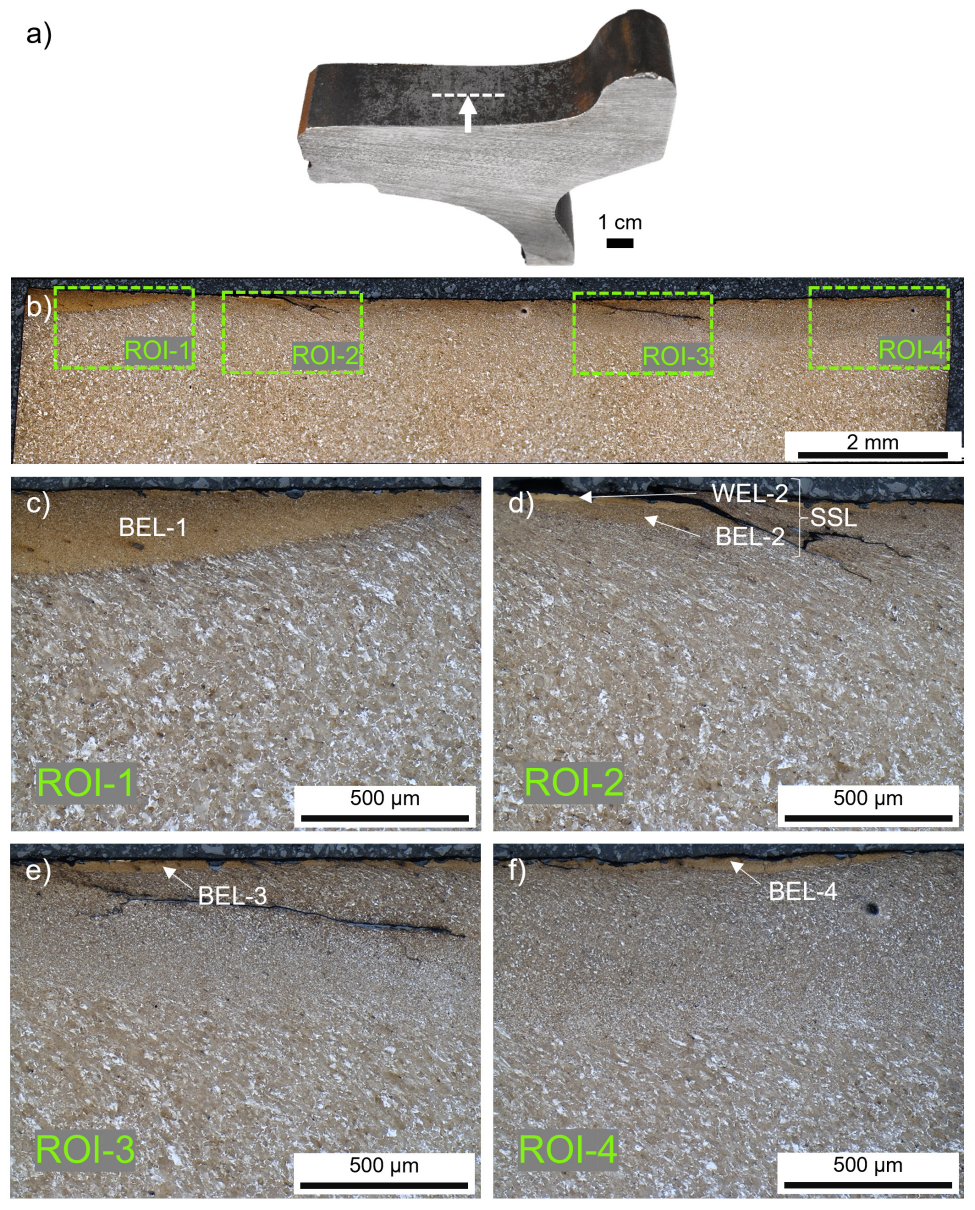
( **a**) The investigated ER7 wheel sample from the field and the related cross-sectional cut ( **b**) in rolling direction. Four different regions of interest (ROI) with white etching layer (WEL)/brown etching layer (BEL) variations are investigated in more detail, optical microscope images are given for ROI-1 ( **c**), ROI-2 ( **d**), ROI-3 ( **e**), and ROI-4 ( **f**).

A rail wheel (0.95 m in diameter) after ~200,000 km in service is provided by the Austrian Federal Railways. The material is a wheel steel with a composition of Fe-0.52C-0.8Mn-0.4Si-0.3Cr-0.3Cu-0.3Ni (in wt%), which is widely used on European railway networks ^26^. The worn tread surface of this wheel is investigated by cutting out a slice (thickness of ~3 mm) of the wheel using a band saw (FMB Pegasus G; FMB s.r.l., Italy) ( Figure 1a). Then, a laboratory cutting device (Struers Secotom-50; Struers ApS, Denmark) is used to cut the surface region of the slice in several cubic samples of ~1x1x1 mm. To create cross-sectional cuts in rolling direction, the cubic samples are embedded in conductive compounds (CitoPress-30, Resing: PolyFast; Struers ApS, Denmark). The embedded samples are then grinded and polished (Tegramin-30; Struers ApS, Denmark) in various steps (SiC Paper #220, MD-Largo 9µm, MD-Dac 3µm and MD-Nap 1µm; Struers ApS, Denmark). Finally, the cross-sectional cuts are etched with diluted nitric acid (3 vol% HNO3, in 97 vol% ethanol for 3 s at room temperature).

Microstructural characterizations are primarily performed by optical microscopy (OM) (Axio Imager M2m, Carl Zeiss AG, Germany) in bright-field mode, images are captured and thicknesses of layers are measured by using IMS Client (Imagic Bildverarbeitung AG, Switzerland). Further, a scanning electron microscopy (SEM) (Jeol JIB 4700F, Jeol Ltd., Japan) is performed, where the secondary electron detector is used to investigate the characteristics of the observed microstructures, 15 kV acceleration voltage is applied. Low-load Vickers hardness measurements are executed using a Future-Tech FM-700 hardness tester (Future-Tech FM-700, Future Tech Corp., Japan) using a load of 0.01 kp (0.098 N). The diagonals of the indents are measured with an OM.

## Results

Along the tread surface of the investigated wheel four specific regions of interest (ROIs) with mechanically and thermally affected microstructures are investigated, see Figure 1b. Discrepancies concerning the degree of etching can be excluded since all the regions are detected on the same cross-sectional sample in the middle of the tread. It can be seen how different the evolved microstructures are within several millimeters. Hence, the local contact situation and the environmental influences vary widely. Within ROI-1 – ROI-4 a brownish-appearing layer can be seen at the current magnification ( Figures 1c, d, e and f), therefore these layers are indicated as BELs. In ROI-2 a stratification containing a white appearing layer (WEL-2) can be observed at the top of BEL-2. This combination is stated as an SSL where severe cracking is evident ( Figure 1d). To identify possible thinner WELs on top of the observed BELs, a more detailed microstructural analysis is presented in the following paragraphs for each ROI.

**Figure 2.**
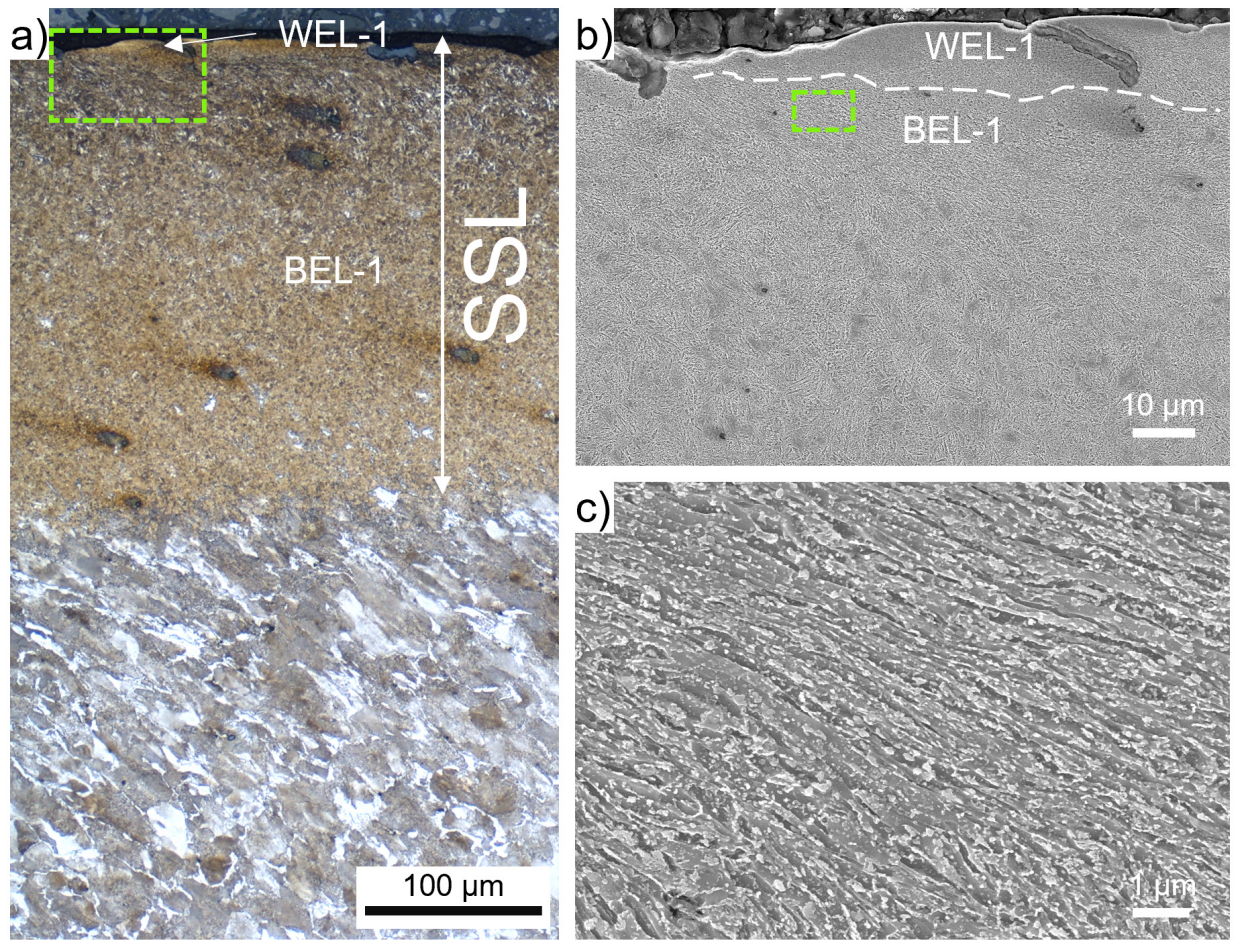
( **a**) shows the evolved microstructure within ROI-1 (see Figure 1b, c) consisting of a thin white etching layer (WEL) at the top, a ~250 µm thick brown etching layer (BEL) underneath, segueing into a deformed ER7 microstructure. ( **b**) presents a scanning electron microscope (SEM) image of the WEL and BEL area framed in ( **a**). A higher magnification SEM image of the BEL-1 microstructure is given in ( **c**).

ROI-1 shows a massive brownish-appearing layer (named BEL-1) with an underlying deformed ferritic-pearlitic microstructure ( Figure 1c). The thickness of the BEL-1 is up to 250 µm, with decreasing extent towards a transition to the deformed wheel material. With increased magnification ( Figure 2a) a bright-appearing thin layer (WEL-1) can be observed on top of the BEL-1. Hence, this region can be stated as SSL, combining a WEL with an underlying BEL. Within the WEL-1 small breakouts can be seen, as well as crack initiation ( Figure 2b), while the average layer thickness is ~20 µm. The morphology of the BEL-1 just underneath the WEL-1 is presented by the SEM image in Figure 2c, showing a severely plastically deformed (SPD) microstructure with an alignment under a certain angle to the surface. Further, globular particles can be identified as randomly distributed. Due to the high degree of deformation, the cementite lamellae of the pearlite of the ER7 microstructure break and thermal activation have led to the spherodization of the cementite lamellae fragments. The low-load hardness measurements executed within the area of the BEL-1 region shown in Figure 2c come up with hardness values of 537±71 HV0.01.

**Figure 3.**
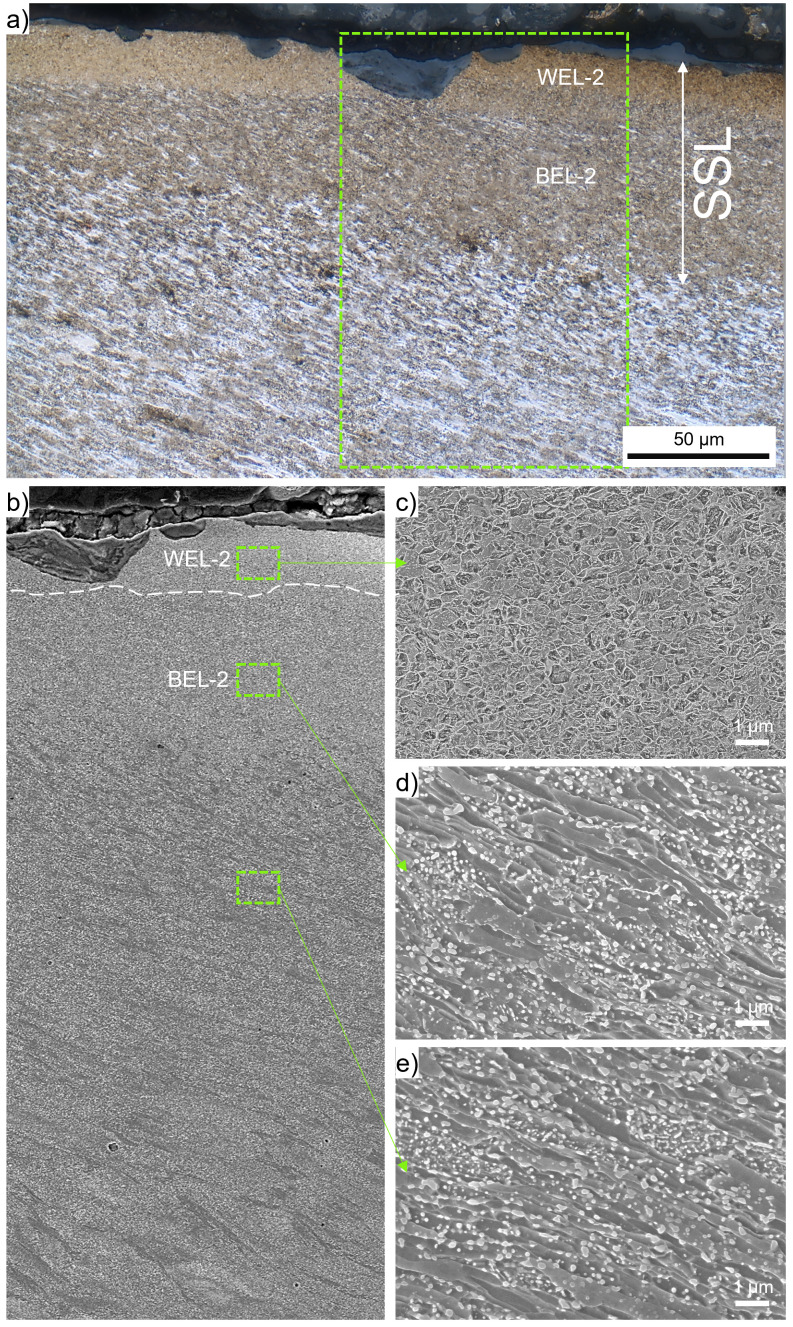
( **a**) shows the stratified surface layer (SSL) (white etching layer-2 (WEL-2) and brown etching layer-2(BEL-2) within ROI-2 (see Figure 1b, d). ( **b**) presents a scanning electron (SEM) image of the region framed in ( **a**). For more details, higher magnification SEM images of certain locations are given: WEL-2 ( **c**), BEL-2 ( **d**), and the underlying deformed ER7 microstructure ( **e**).

Within ROI-2, an SSL can be seen in Figure 1d, accompanied by a crack initiation and propagation along an angle similar to the shear-deformed near-surface microstructure into depth. The SSL is shown by higher magnification OM in Figure 3a. The SSL consists of an almost featureless topmost layer with a thickness of ~30 µm (indicated as WEL-2) and an underlying brownish-appearing layer with a thickness of ~50µm, designated as BEL-2. Micro-spalling can be seen in the WEL-2, a SE image of the SSL is given in Figure 3b. To analyze the different microstructural characteristics, high magnification SEM images are presented: Figure 3c shows the microstructure of WEL-2, coming up with a fine mesh-like structure without any preferred orientation detectable. The image indicates some nanometer-sized globular particles fine dispersed. Hardness values of 1184±28 HV0.01 are obtained within this region. In the region BEL-2 ( Figure 3d), a coarser deformed microstructure aligned under an angle of ~30° to the surface can be identified, containing some globular particles with significantly larger proportions with respect to the WEL-2. The low load hardness measurements reveal results of 500±42 HV0.01. Comparable microstructure can be seen in the microstructure underneath the BEL-2 ( Figure 3e), but with a slight change in the alignment angle of the deformed microstructure. Comparable hardness values are determined with 521±47 HV0.01. The globular particles are suggested to result from broken cementite lamellae (originating from the pearlite of the ER7 wheel microstructure), spherodized due to thermal influences.

**Figure 4.**
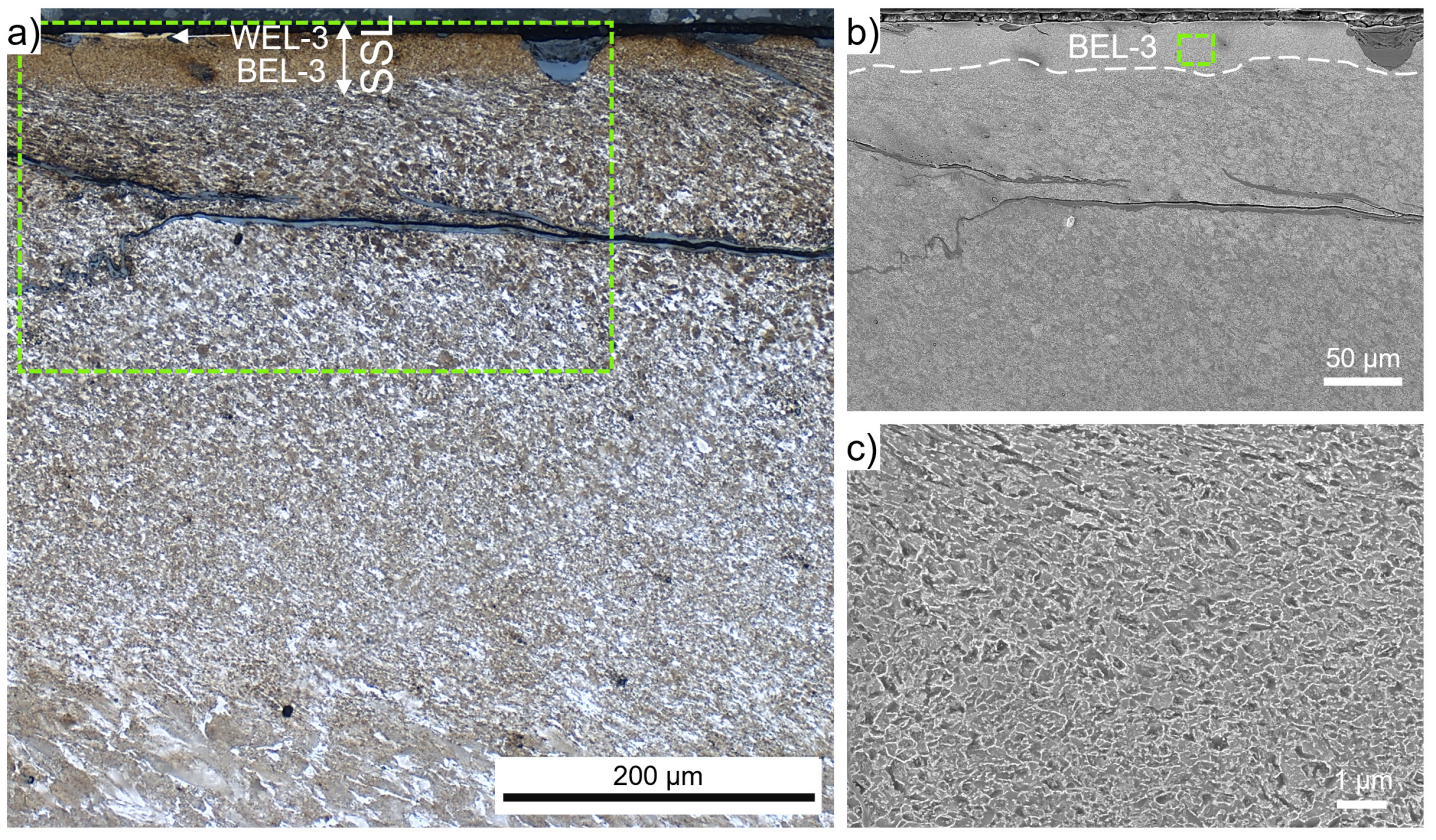
( **a**) shows the evolved microstructure within ROI-3 (see Figure 1b, e), where a very thin white etching layer (WEL) is on top of a brown etching layer (BEL) with a thickness of ~50 µm (BEL-3). This layer is situated on a severely plastically deformed (SPD) microstructure accompanied by severe cracking. ( **b**) presents a scanning electron microscope (SEM) image of the WEL and BEL area framed in ( **a**). A higher magnification SEM image of the BEL-3 microstructure is given in ( **c**).

The brownish-appearing layer within ROI-3, where a severe crack network is visible ( Figure 1e), is analyzed in more detail by higher magnification OM and SEM ( Figure 4). The near-surface microstructure shows parts of a white appearing thin layer on top of the BEL, named WEL-3 ( Figure 4a). Crack initiation can be seen along the surface, propagating into a brownish appearing layer with a thickness of ~50 µm (BEL-3). On the right side of the SE image ( Figure 4b) a spalled BEL region can be detected. The cracks initiating at the surface seem to stop at the interface of the BEL-3 to the underlying SPD microstructure. However, severe horizontal cracks are observed at a depth of ~100 – 200 µm from the surface. The SPD microstructure gradually changes to a deformed and aligned ER7 microstructure with increasing grain sizes. The focus of this study, the microstructure of the BEL, is further investigated by high magnification SE images presented in Figure 4c. A randomly orientated mesh-like microstructure can be seen, with a certain degree of spheroidization. The low load Vickers hardness measurements in this area show hardness values of 509±24 HV0.01.

**Figure 5.**
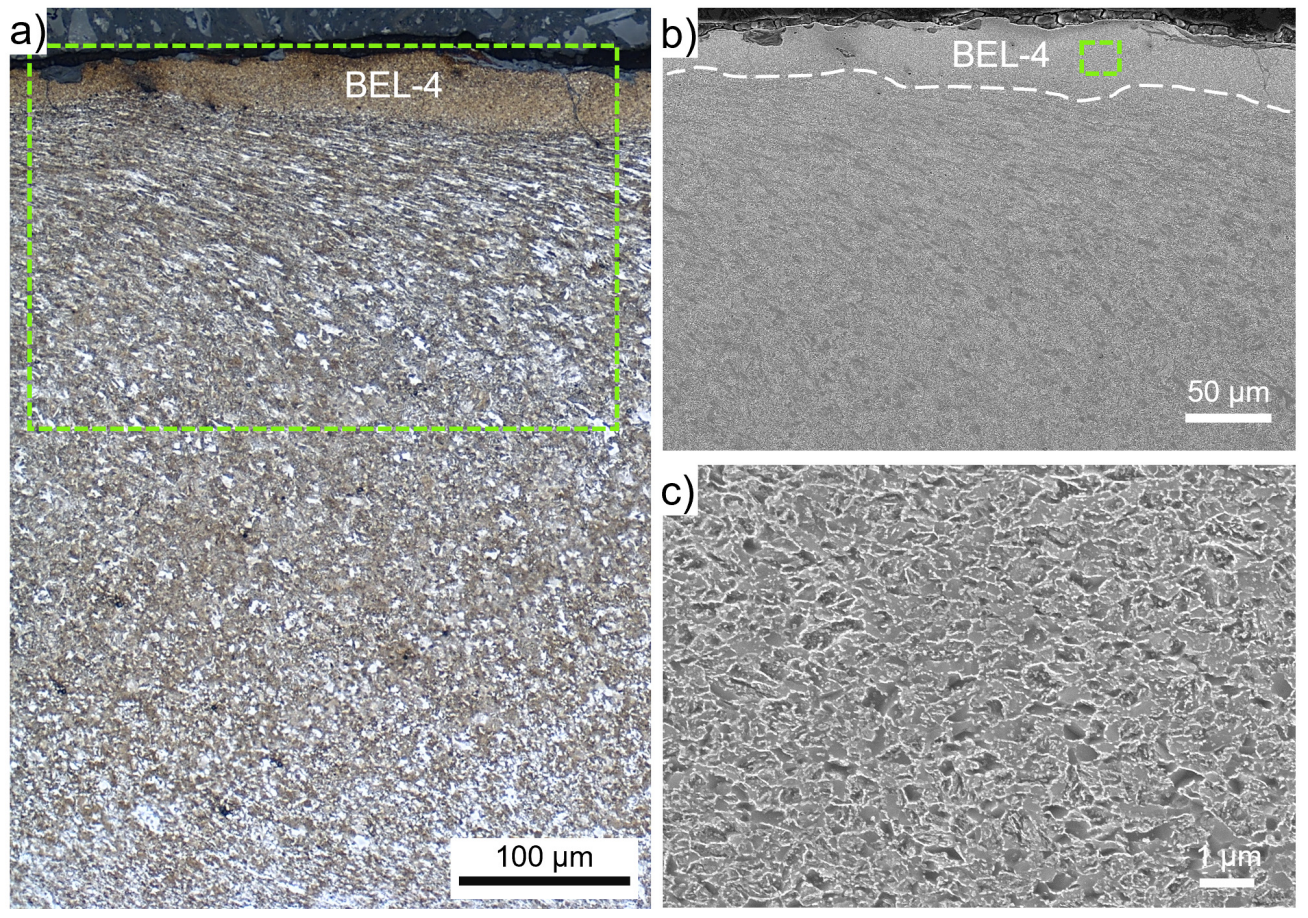
( **a**) shows the microstructure within ROI-4 (see Figure 1b, f), where a brown etching layer (BEL) with a thickness of ~50 µm (BEL-4), is situated on an severely plastically deformed microstructure. ( **b**) presents a scanning electron microscope (SEM) image of the WEL area framed in ( **a**). A higher magnification SEM image of the WEL-4 microstructure is given in ( **c**).

The layer observed at the wheel surface in ROI-4 is appearing brownish and therefore designated as BEL ( Figure 1f). in contrast to the other BELs observed, no WEL can be identified on top of the BEL-4. Micro-spalling, crack initiation, and crack propagation through the WEL under almost 90° to the surface can be observed ( Figure 5a), the layer is named WEL-4. The alignment of the underlying ER7 microstructure is visible ( Figures 5a and b). The microstructure within WEL-4 is shown by SE imaging in Figure 5c, indicating a fine-grained mesh-like structure. Further, a certain degree of spherodization can be identified. The results of the hardness measurements come up with pronounced scattering, howsoever, high hardness values are indicated with 979±141 HV0.01.

## Discussion

The characterization of different evolved near-surface microstructures on a well-established rail wheel from service outpoints the wide range of microstructural variations with respect to the location along the tread surface. The naming WEL and BEL is originally based on the appearance under the OM after etching with Nital, hence, based on the degree of etching and the image settings. Therefore, a common naming based on OM images is hardly possible and questionable. The information of the characterization by SEM and hardness measurements may enable a more consistent naming. The results within this work show the WEL as a fine-grained mesh-like microstructure without globular particles and high hardness (~1000 HV0.01), see WEL-2. In some cases, even the brownish appearing regions can exhibit hardness values above 900 HV0.01 (see BEL-4), but a more dissolved microstructure with spheroidization. The definition is in this case arguable, since no WEL can be detected on top of the BEL, in contrast to the other regions investigated. But, the BEL-3 shows comparable microstructure in the SEM, with lower hardness (~500 HV0.0.1). This demonstrates the demand of further in-depth analysis to understand the microstructural characteristics and therefore the formation process and naming. In general, evolved microstructures with hardness in the range of 400 – 600 HV0.01, brownish appearance in the LOM, and pronounced spherodized cementite lamellae leading to globular particles can be indicated as BEL. It is shown, that even within a BEL the microstructure can locally differ, see BEL-1. In most cases, when observing a BEL, a WEL can also be identified, representing an SSL. The thicknesses of the WEL and BEL is varying, hence, the SSL often is detectable only with higher magnification, see ROI-1 and ROI-3. Samples from field are worn and the analysis is always a short snapshot, which additionally complicates a common identification and classification. 

## Conclusions

The work shows the broad variety of evolved near-surface microstructures on rail wheels, which makes a common terminology hardly possible, however, with increasing numbers of published studies certain comparison is possible. A rough classification can be made for the examined wheel steel (Fe-0.52C-0.8Mn-0.4Si-0.3Cr-0.3Cu-0.3Ni):

▪ A so-called WEL appears white in OM after etching with Nital, shows a fine mesh-like microstructure without globular particles in SEM, and hardness in the range of 900 – 1200 HV0.01
▪ The BEL looks brownish in OM after etching with Nital, shows pronounced globular particles (spherodized cementite lamellae) within SPD microstructure in SEM, and hardness in the range of 400 – 600 HV0.01 (with variations up to 900 HV)

In most cases, a BEL is always accompanied by a WEL, forming a so-called SSL. Crack initiation and crack networks are observed in the presence of SSLs on the ER7 wheel from service, indicating a relation to fatigue crack initiation and possible failure of the wheel.

## Data Availability

Zenodo, Microstructural characterization of near-surface microstructures on rail wheels in service – an insight into "stratified surface layers": Supplementary Data. https://doi.org/10.5281/zenodo.7836670 ^27^. This project contains the following underlying data: fig_ORE_Freisinger (1).JPG fig_ORE_Freisinger (1).tif fig_ORE_Freisinger (10).jpg fig_ORE_Freisinger (10).tif fig_ORE_Freisinger (2).jpg fig_ORE_Freisinger (2).tif fig_ORE_Freisinger (3).jpg fig_ORE_Freisinger (3).tif fig_ORE_Freisinger (4).jpg fig_ORE_Freisinger (4).tif fig_ORE_Freisinger (5).jpg fig_ORE_Freisinger (5).tif fig_ORE_Freisinger (6).jpg fig_ORE_Freisinger (6).tif fig_ORE_Freisinger (7).jpg fig_ORE_Freisinger (7).tif fig_ORE_Freisinger (8).jpg fig_ORE_Freisinger (8).tif fig_ORE_Freisinger (9).jpg fig_ORE_Freisinger (9).tif hardness_ORE_Freisinger.xlsx Data are available under the terms of the Creative Commons Attribution 4.0 International license (CC-BY 4.0).
