# Supplementary material for: Microstructural characterization of near-surface microstructures on rail wheels in service – an insight into “stratified surface layers”
Source: Open Res Eur. 2023 Jul 3;3:73. Originally published 2023 May 2. [Version 2] doi: 10.12688/openreseurope.15881.2 (PMC10445912; doi:10.12688/openreseurope.15881.2)
Supplement: Peer review history

# Peer review: Reviewer response for version 2

**DOI:** 10.21956/openreseurope.17524.r33449

**Published:** 2023-07-10

**Authors:** Roumen H. Petrov

**Affiliations:** 1 Department of Electromechanical, Systems and Metal Engineering, Universiteit Gent, Ghent, Flanders, Belgium

The authors did their best to implement the requested changes. Now the submission is enough good to be indexed. It provides interesting results and can be a good base for further in-depth studies of the phenomena WEL/BEL on wheels.

 One minor comment:

- In the description of the etching technique please replace  "HNO3" with "HNO _3 _"; The symbol in the subscript are used to show atomic percents which is the correct description of the nitric acid.

Is the study design appropriate and does the work have academic merit?

Partly

Is the work clearly and accurately presented and does it cite the current literature?

Yes

If applicable, is the statistical analysis and its interpretation appropriate?

Not applicable

Are all the source data underlying the results available to ensure full reproducibility?

No

Are the conclusions drawn adequately supported by the results?

No

Are sufficient details of methods and analysis provided to allow replication by others?

Partly

Reviewer Expertise:

Micro structural characterization of damage and fracture. Optical microscopy, Scanning and transmission Electron Microscopy, XRD, texture , EBSD, TKD, ACOM -TEM, 3D-EBSD. Damage on rails and bearings. Metals technology-thermal and thermo-chemical treatment;

I confirm that I have read this submission and believe that I have an appropriate level of expertise to confirm that it is of an acceptable scientific standard.

# Peer review: Reviewer response for version 1

**DOI:** 10.21956/openreseurope.17155.r31402

**Published:** 2023-05-30

**Authors:** Roumen H. Petrov

**Affiliations:** 1 Department of Electromechanical, Systems and Metal Engineering, Universiteit Gent, Ghent, Flanders, Belgium

The paper is interesting and shows nice metallographic microstructures on the rail wheel. The number of papers that characterize the WEL on wheels is not very large and this work could be an interesting contribution, provided that it corrects several important elements. I am afraid that I cannot recommend the paper for indexing as it is. The authors must re-work it and show very clear their message. Additional characterizations and the use of high-resolution characterization techniques are required in order to understand better the WEL and BEL microstructures on these steels.

 A few more detailed comments follow:

1. I advise the authors not to use directly the abbreviation ER7 without explanation. ER7 Material 650mm Railway Wagon Wheels AAR Standard is known only by specialists in the field but not by general metallurgists.
2. The sentence: “Finally, the cross-sectional cuts are etched with diluted nitric acid (3% HNO3, 97% ethanol).” Should be: “Finally, the cross-sectional cuts are etched with diluted nitric acid (3vol% HNO3, in 97vol% ethanol for XXX s at room temperature).” It is of critical importance to mention the exact etching conditions, because all the discussions in this work are based on the differences in the appearance of the microstructure. This appearance is critically dependent on the way of sample preparation and especially the etching technique.
3. What is “...load of 0.01 kp”? kp is not mentioned in any standards. It should be 10gf (gram force) which is much more understandable.

Comments on the results:

1. The observed results are critically dependent on the loading conditions which were not given. It is written: “…with a milage of ~200,000 km.” It is not enough to mention only the mileage. The load should be mentioned as well because it is an important part of the damage formation.  Additionally, the friction conditions determine the temperature changes in the contact zones. They should be known in order to have a meaningful observation of the formation of these structures in the wheel.  
2. The characterization of the microstructures is based mainly on SEM and OM  but does it represents all possible microstructures that can form in the damaged wheel? It is well known that WEL in rails is a mixture of ultrafine-grained martensitic structures and retained austenite (RA). Here the authors did not mention any RA. Finally, is there are differences between the WEL on the rail and the steel?
3. In Fig. 5a the authors show WEL which is actually brown? Why do they call it white? Its microstructure is exactly the same as the microstructure of the BEL shown in Fig. 4. (compare Fig.4c and Fig.5c). In the cited works [22] and [23] and [25] the differences between the BEL and WEL are very clear and they appear indeed in the white and brown colors, not in the shades of brown. To understand better the nature of the formed microstructures additional X-ray diffraction should be carried out and if possible EBSD.

Is the study design appropriate and does the work have academic merit?

Partly

Is the work clearly and accurately presented and does it cite the current literature?

Yes

If applicable, is the statistical analysis and its interpretation appropriate?

Not applicable

Are all the source data underlying the results available to ensure full reproducibility?

No

Are the conclusions drawn adequately supported by the results?

No

Are sufficient details of methods and analysis provided to allow replication by others?

Partly

Reviewer Expertise:

Micro structural characterization of damage and fracture. Optical microscopy, Scanning and transmission Electron Microscopy, XRD, texture , EBSD, TKD, ACOM -TEM, 3D-EBSD. Damage on rails and bearings. Metals technology-thermal and thermo-chemical treatment;

I confirm that I have read this submission and believe that I have an appropriate level of expertise to state that I do not consider it to be of an acceptable scientific standard, for reasons outlined above.

# Response

**Published:** 2023-06-19

**Authors:** Matthias Freisinger

**Affiliations:** AC2T research GmbH, Austria

We highly appreciate your review based on your excellent scientific background in this topic and hope that we meet your comments in the revised article. We try to sharpen our message of the article – as it presents an overview of evolved layers formed on rail wheels in service (with unknown loading history). Further, we are pleased you recognize the modest number of papers on WELs on wheels and the contribution of our article. Additional in-depth characterisations of evolved WELs and BELs are of high interest, however, due to the numerous variations of the evolved microstructures this would go beyond the scope of the current article. In the following section, we want to give answers to your valuable comments of the review:  

1. Thank you for this comment, we see this issue and changed the wording without using the “ER7” abbreviation.
2. We highly appreciate your comment regarding etching procedure. In fact, this is crucial since the current analysis is solely based on optical microscopy appearance. Further, in-depth analysis is of great interest, but not aim of this overview article.
3. Based on our knowledge, Vickers hardness measurements are defined by the applied Kilopond (kp), however, since this is a non-standard gravitational metric unit of force, we specified the testing parameters in Newtons (N) in the revised manuscript.
4. In fact, the loading conditions are critical on the formation of evolved microstructures on rail wheels. Unfortunately, it is practically impossible to know the detailed mechanical and thermal loading history of samples from the field. This is the case in the analysed sample in this article as well. Hence, we cannot give better loading parameters. However, the article should show a rough overview about the evolved microstructural layers. To study these layers systematically, a lab approach with defined loading parameters is vital, hence, the authors are presenting such a lab approach at the ECOTRIB conference in Bari (IT) in June 2023. Further, a manuscript is in progress on the fatigue behaviour of defined WELs and BELs formed by reproducible thermal and mechanical loadings in the lab on wheel steels.
5. Thanks for this comment. The current article is indeed restricted on SEM and OM. Due to the vast variety of WELs and BELs more detailed analysis would be preferable for more detailed results. However, the aim of this article is a rough overview, and the etching analysis shows the desired microstructural changes. The current work does not claim a detailed description of formation (detection of RA,…). Differences between WEL on rails and wheels are not studied, which would require a comparison of numerous variations detected on rails and wheels and would go beyond the scope of the submitted article.
    
6. When revising the article, we decided to name this BEL, based on the comparable microstructure. Moreover, we sharpened the discussion, and we hope we address your comment. Advanced analysis would be needed, but, beyond the scope of this overview work.

# Peer review: Reviewer response for version 1

**DOI:** 10.21956/openreseurope.17155.r31786

**Published:** 2023-05-25

**Authors:** Christoph Gammer

**Affiliations:** 1 Erich Schmid Institute of Materials Science, Austrian Academy of Sciences, Leoben, Austria

This article analyzes the near-surface microstructure of a rail wheel after service. The white etching layer (WEL) and brown etching layer (BEL) forming the stratified surface layer (SSL) are identified. In addition to detailed optical microscopy after Nital etching, microhardness measurements and scanning electron microscopy are preformed from the WEL and BEL. Finally, cracks are shown in the SSL.

 The manuscript is motivated, well written and the microstructure is clearly described. In addition, the results are discussed with respect to general problems of accurately describing microstructures in rail wheels.

 A small typos should be fixed (HV0.0.1) and care should be taken that all abbreviations are introduced (SE). In addition, it would help readers that are not clearly familiar with railway wheels to include some schematic in Figure 1 showing how the piece was extracted from an actual wheel.

Is the study design appropriate and does the work have academic merit?

Yes

Is the work clearly and accurately presented and does it cite the current literature?

Yes

If applicable, is the statistical analysis and its interpretation appropriate?

Yes

Are all the source data underlying the results available to ensure full reproducibility?

Yes

Are the conclusions drawn adequately supported by the results?

Yes

Are sufficient details of methods and analysis provided to allow replication by others?

Yes

Reviewer Expertise:

Transmission electron microscopy, Metallic Glasses, Nanostructured Materials

I confirm that I have read this submission and believe that I have an appropriate level of expertise to confirm that it is of an acceptable scientific standard.

# Response

**Published:** 2023-06-19

**Authors:** Matthias Freisinger

**Affiliations:** AC2T research GmbH, Austria

We want to thank you for your thorough review and the approval of our article to show relevant results on the analysis of evolved microstructural regions on rail wheels during service. Revision on typos and minor changes based on your comments are done.
